# First instar larvae of endemic Australian Miltogramminae (Diptera: Sarcophagidae)

**DOI:** 10.1038/s41598-020-80139-x

**Published:** 2021-01-29

**Authors:** Krzysztof Szpila, Kinga Walczak, Nikolas P. Johnston, Thomas Pape, James F. Wallman

**Affiliations:** 1 Department of Ecology and Biogeography, Faculty of Biological and Veterinary Sciences, Nicolaus Copernicus University, Lwowska 1, 87-100 Toruń, Poland; 2 Centre for Modern Interdisciplinary Technologies, Nicolaus Copernicus University, Wileńska 4, 87-100 Toruń, Poland; 3 Centre for Sustainable Ecosystem Solutions, School of Earth, Atmospheric and Life Sciences, University of Wollongong, Wollongong, NSW 2522 Australia; 4 Natural History Museum of Denmark, University of Copenhagen, Universitetsparken 21, 2100 Copenhagen Ø, Denmark; 5 School of Life Sciences, University of Technology Sydney, PO Box 123, Broadway, NSW 2007 Australia

**Keywords:** Evolution, Zoology

## Abstract

The first instar larva of a species of the Australian endemic genus *Aenigmetopia* Malloch is described for the first time, along with the first instar larvae of three other Australian species representing the genera *Amobia* Robineau-Desvoidy and *Protomiltogramma* Townsend. Larval morphology was analysed using a combination of light microscopy, confocal laser scanning microscopy and scanning electron microscopy. The following morphological structures are documented: pseudocephalon, antennal complex, maxillary palpus, facial mask, modifications of thoracic and abdominal segments, anal region, spiracular field, posterior spiracles and details of the cephaloskeleton. Substantial morphological differences are observed between the three genera, most notably in the labrum and mouthhooks of the cephaloskeleton, sensory organs of the pseudocephalon, spinulation, sculpture of the integument and form of the spiracular field. The first instar larval morphology of *Aenigmetopia amissa* Johnston, Wallman, Szpila & Pape corroborates the close phylogenetic affinity of *Aenigmetopia* Malloch with *Metopia* Meigen, inferred from recent molecular analysis. The larval morphology of *Amobia auriceps* (Baranov), *Protomiltogramma cincta* Townsend and *Protomiltogramma plebeia* Malloch is mostly congruent with the morphology of Palaearctic representatives of both genera.

## Introduction

The first larval instar of Miltogramminae show diverse and peculiar morphologies, which most likely reflect adaptations to locating suitable food sources by digging through sand, often combined with different types of kleptoparasitism^1^. During the past decade much information about the larvae of this subfamily has been accumulated e.g.^2–4^, resulting in the availability of morphological data for almost all Palaearctic genera. In contrast to this, the preimaginal instars of most non-Palaearctic taxa remain unknown, and the Australasian region stands out in that morphological information on the immature stages of its miltogrammine fauna is completely lacking.

Subfamily Miltogramminae is a relatively diverse group with more than 600 described species^5,6^. Most of the known species occur in dry habitats, particularly the deserts and semideserts of Asia and Africa^7^. Recent targeted taxonomic research has revealed a remarkable miltogrammine diversity in Australia (currently 28 named species), with the most speciose genera being *Protomiltogramma* Townsend (12 spp.) and *Aenigmetopia* Malloch (5 spp.), while the genera *Amobia* Robineau-Desvoidy (3 spp.)*, Macronychia* Rondani (1 sp.)*, Metopia* Meigen (2 spp.), *Miltogramma* Meigen (4 spp.) and *Senotainia* Macquart (1 sp.) are more modestly represented^8–12^. The Australian fauna constitutes only a small part of the global miltogrammine diversity^6^, with a noteworthy high proportion for *Protomiltogramma* (32.4% of global diversity), modest for *Amobia* (21.4%), and very low for the other genera: *Macronychia* (3.8%), *Metopia* (4.9%), *Miltogramma* (3.4%) and *Senotainia* (1.5%)^6–14^,. However, endemism is high (89.3%) as only three species, one in each of *Amobia*, *Metopia* and *Senotainia*, are found outside Australia.

The present study fills an important gap in the morphospace of a large taxon of Diptera by providing the first morphological documentation of the preimaginal stages of four Australian miltogrammine species: (1) endemic *Aenigmetopia amissa* Johnston, Wallman, Szpila & Pape, *Protomiltogramma cincta* Townsend, *Protomiltogramma plebeia* Malloch; and (2) *Amobia auriceps* (Baranov), widely distributed in the Oriental and Australasian-Oceanian regions. The newly discovered larval morphology of Australian species is compared with existing data across the global fauna of Miltogramminae. We also discuss conflicts and congruence between larval morphology and both former and current phylogenetic hypotheses presented for the genera in question. Special focus is on the systematic position of *Aenigmetopia*, the only endemic genus of Australian miltogrammines.

## Results

The habitus of the first instar of *Ae. amissa*, *Am. auriceps*, *P. cincta* and *P. plebeia* follows the general pattern for the Calyptratae, with the body being divided into a bilobed pseudocephalon (pc) equipped with antennal and maxillary sensory organs, three thoracic segments (t1–t3), seven abdominal segments (a1–a7), and an anal division (ad) carrying the posterior spiracles (ps) (Fig. 5A,B,E,F).

**Figure 1:**
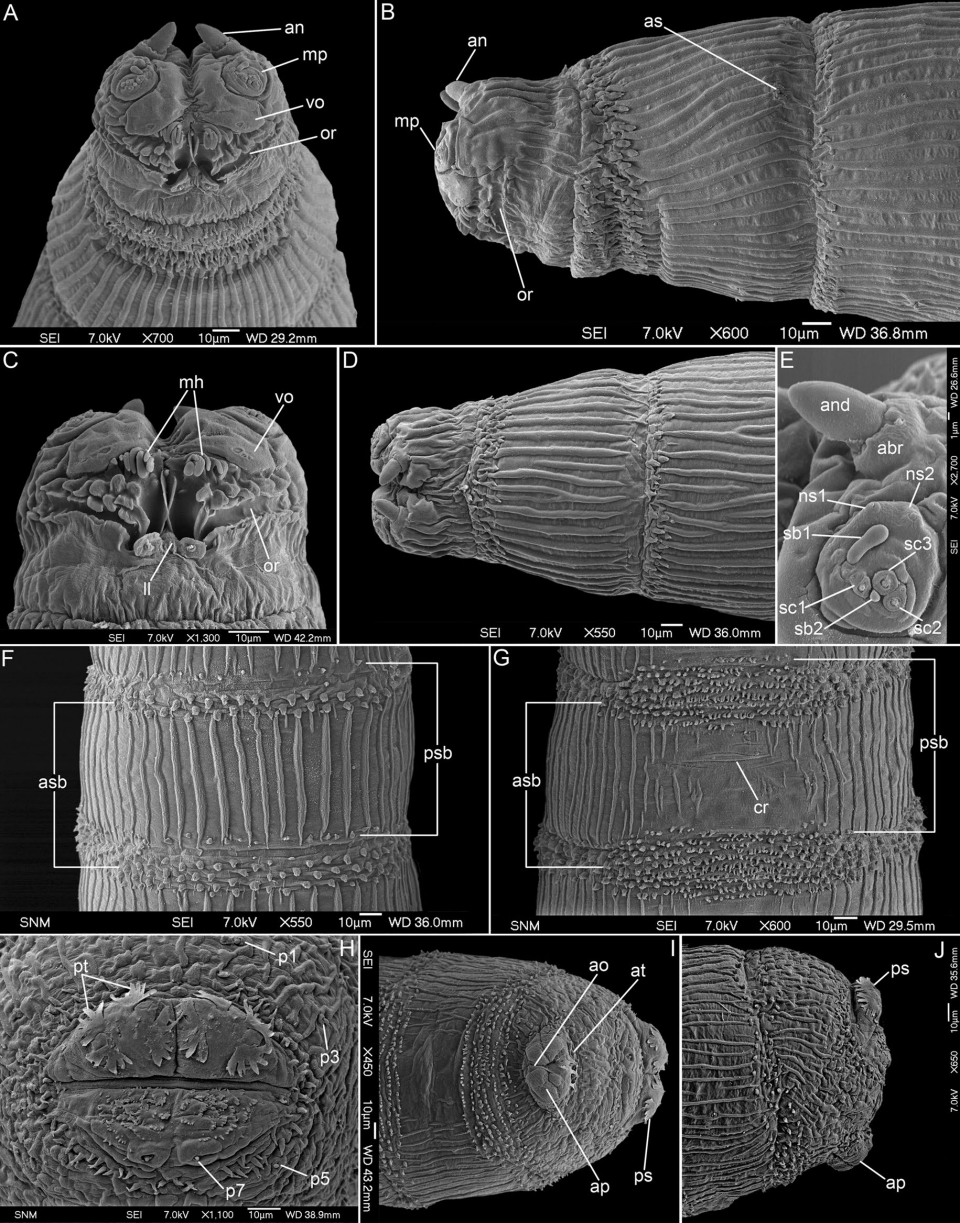
First instar larva of *Aenigmetopia amissa*. (**A**) Anterior end, ventral view. (**B**) Anterior end, lateral view. (**C**) Pseudocephalon, ventral view. (**D**) Anterior end, dorsal view. (**E**) Antennal complex and maxillary palpus. (**F**) First abdominal segment, dorsal view. (**G**) Second abdominal segment, ventral view. (**H**) Anal division, spiracular field. (**I**) Posterior end, ventral view. (**J**) Posterior end, lateral view. abr, antennal basal ring; an, antennal complex; and, antennal dome; ao, anal opening; ap, anal papilla; as, anterior spiracle; asb, anterior spinose band; at, anal tuft; cr, transverse crevice; ll, labial lobe; mh, mouthhook; mp, maxillary palpus; ns1, first additional sensillum coeloconicum; ns2, second additional sensillum coeloconicum; or, oral ridges; p1–p7, papillae around spiracular field; ps, posterior spiracle; psb, posterior spinose band; pt, peristigmatic tufts; sb1–3, sensilla basiconica 1–3; sc1–3, sensilla coeloconica 1–3; vo, ventral organ.

*Aenigmetopia amissa* Johnston, Wallman, Szpila & Pape, 2020.

(Figs. 1A–J, 5A,C, 6A,B).

Material examined: 17 larvae from a single female: /Australia, NT, 585/Litchfield NP/Lost City/-13.21652 130.73544/10 Nov 2017/leg. FG-NT Expedition/.

*Pseudocephalon*. Antennal complex (an) large, antennal dome (and) oval but with slightly conical tip, antennal basal ring (abr) high (Fig. 1A,E); maxillary palpus (mp) shaped as a flat disc clearly distinguished from surrounding cuticle, first sensillum basiconicum (sb1) long with slightly swollen tip and slightly shifted away from central cluster of sensilla toward medio-dorsal border of palpus, sensilla ns1–2 situated on dorsal periphery of maxillary palpus (Fig. 1A,E); ventral organ (vo) on flat, fleshy lobe (Fig. 1A,C); oral ridges (or) visible as grooves with anterior edge formed by double row of irregular processes (Fig. 1A,C); pseudocephalon behind antennal complex with cuticular ridges (Fig. 1B,D).

*Cephaloskeleton*. Labrum (lb) elongated, slender anterior part of labrum shorter than broader basal part, anterior part of labrum curved downward and with pointed tip (Figs. 5C, 6A); mouthhook (mh) slightly curved, basal part with lateral arm (Figs. 5C, 6A,B), tip of mouthhook with row of 6–7 curved teeth of different size, tips of teeth semicircular (Fig. 1C); intermediate sclerite (is) slightly below parastomal bars (pb) in lateral view (Figs. 5C, 6A,B); parastomal bars (pb) moderately long (Figs. 5C, 6A,B); vertical plate (vp) with similar width like ventral and dorsal cornua (dc and vc) (Figs. 5C, 6A); dorsal bridge absent.

*Thoracic segments*. Anterior spinose bands (asb) with from 3–4 (dorsally) to 11–12 (ventrally on t1) rows of spines, spines arranged separately from each other, mono- or rarely bi-cuspid (Fig. 1A,B,D); t1 laterally with aperture of anterior spiracle (as) (Fig. 1B); remaining area of t1–t3 with densely set cuticular ridges (Fig. 1A,B,D); Keilin’s organ with slightly elongated sensilla.

*Abdominal segments*. Anterior spinose bands (asb) on a1–a7 with from 1–2 to 8–9 rows of spines, all bands complete (Fig. 5A), posterior spinose band (psb) on all segments complete but narrow with 1–2 rows of spines, spines small and arranged separately from each other, with ventral spines smaller than lateral and dorsal spines and arranged in short irregular rows (Fig. 1F,G); lateral creeping welts (lcw) developed and covered by spines; all abdominal segments with regular cuticular ridges on entire surface except the midventral area (Fig. 1G,I).

*Anal division*. Anterior spinose band on anal division (ad) incomplete, without spines on the middorsal surface (Fig. 1J); anal division except for spiracular field with irregular cuticular ridges and warts (Fig. 1I,J); papillae around spiracular field small and visible as flat protuberances with an apical sensillum (Fig. 1H); spiracular field partly ringed by sparse circle of spines; below posterior spiracle transverse group of small spines directed toward anterior end of body, spines arranged in short clusters where spines are fused basally (Fig. 1H); posterior spiracles (sp) with four peristigmatic tufts (pt) each with numerous (5–7) branches (Fig. 1H); anal papillae rounded (Fig. 1I,J); anal tuft (at) with several spines (Fig. 1I).

**Figure 2:**
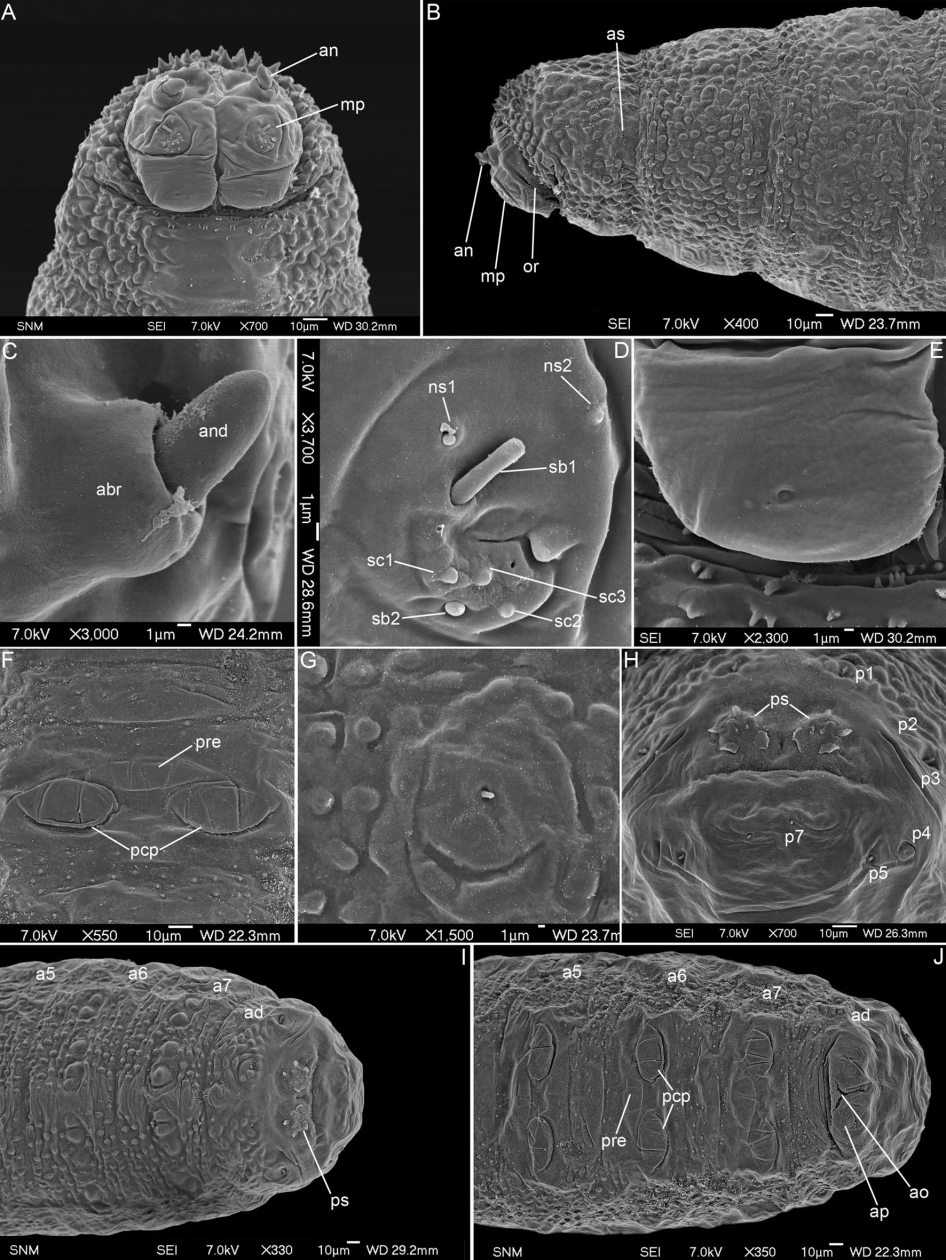
First instar larva of *Amobia auriceps*. (**A**) Anterior end, ventral view. (**B**) Anterior end, lateral view. (**C**) Antennal complex. (**D**) Maxillary palpus. (**E**) Ventral organ. (**F**) Fifth abdominal segment, ventral view. (**G**) First abdominal segment, lateral papilla. (**H**) Anal division, spiracular field. (**I**) Posterior end, dorsal view. (**J**) Posterior end, ventral view. a5–a7, abdominal segments a5–a7; abr, antennal basal ring; an, antennal complex; ad, anal division; and, antennal dome; ao, anal opening; ap, anal papilla; as, anterior spiracle; mp, maxillary palpus; ns1, first additional sensillum coeloconicum; ns2, second additional sensillum coeloconicum; or, oral ridges; p1–p7, papillae around spiracular field; pcp, post-crevice protuberance; pre, pre-crevice protuberance, ps, posterior spiracle; sb1–3, sensilla basiconica 1–3; sc1–3, sensilla coeloconica 1–3.

*Amobia auriceps* (Baranov, 1935).

(Figs. 2A–J, 5B,D, 6C,D).

Material examined: 18 larvae from a single female: /Australia, NT/Litchfield NP/Lost City/-13.190533, 130.774565/10 Nov 2017/leg. FG-NT Expedition/.

*Pseudocephalon*. Antennal complex (an) large, antennal dome (and) oval but with rounded tip, antennal basal ring (abr) high (Fig. 2A,C); maxillary palpus (mp) shaped as a flat disc, first sensillum basiconicum (sb1) very long and slightly shifted away from central cluster of sensilla toward dorsal border of palpus, sensilla ns1 situated on dorsal and ns2 on lateral periphery of maxillary palpus (Fig. 2D); ventral organ (vo) on large flat, fleshy lobe (Fig. 2E); oral ridges (or) present (Fig. 2B); pseudocephalon without ridges or warts (Fig. 2A,B).

*Cephaloskeleton*. Labrum (lb) long, curved downward, gradually tapering to pointed anterior tip (Figs. 5D, 6C); basal part of mouthhook broad, anterior part slender and partly unsclerotised, tip of mouthhook pointed as single tooth (Fig. 6C); intermediate sclerite (is) slightly below parastomal bars (pb) in lateral view (Figs. 5D, 6C,D); parastomal bar (pb) long (Figs. 5D, 6C); vertical plate (vp) broader than width of dorsal and ventral cornua (Fig. 6C); dorsal bridge absent.

*Thoracic segments*. Except for t1, anterior spinose bands difficult to differentiate from warts of interband surfaces, ventral surface of segments smooth without spines or warts (Fig. 2A,B).

*Abdominal segments*. Spines of dorsal and lateral spinose bands gradually reshaped to cuticular warts along each segment, especially on a4–a7; ventral spines smaller than lateral and dorsal ones (Fig. 2F); segments with dorsal and dorsolateral cuticular warts (Figs. 2I, 5B); longitudinal strip of warts also present ventrolaterally on all abdominal segments (Figs. 2J, 5B); six large triangular cones set in a transverse row dorsally, and two similar but smaller cones dorsolaterally on all segments (Figs. 2I, 5B); abdominal segments ventrally with system of prolegs/locomotory protuberances, one protuberance (pre) situated anteriorly to transverse crevice, two others (pcp) situated posteriorly to crevice (Fig. 2F,J); complex of lateral papilla as large, flat field with smooth surface and centrally situated sensillum basiconicum (Fig. 5G).

*Anal division*. Anterior spinose band incomplete, dorsal and dorsolateral spines replaced by warts (Fig. 2I,J); papillae p1–p7 around spiracular field slightly protruded (Fig. 2H); ring of spines around spiracular field absent (Fig. 2H); posterior spiracles (ps) with four small peristigmatic tufts having lobes without incisions (Fig. 2H); anal papillae (ap) rounded (Fig. 2J); spines of anal tuft absent (Fig. 2J).

**Figure 3:**
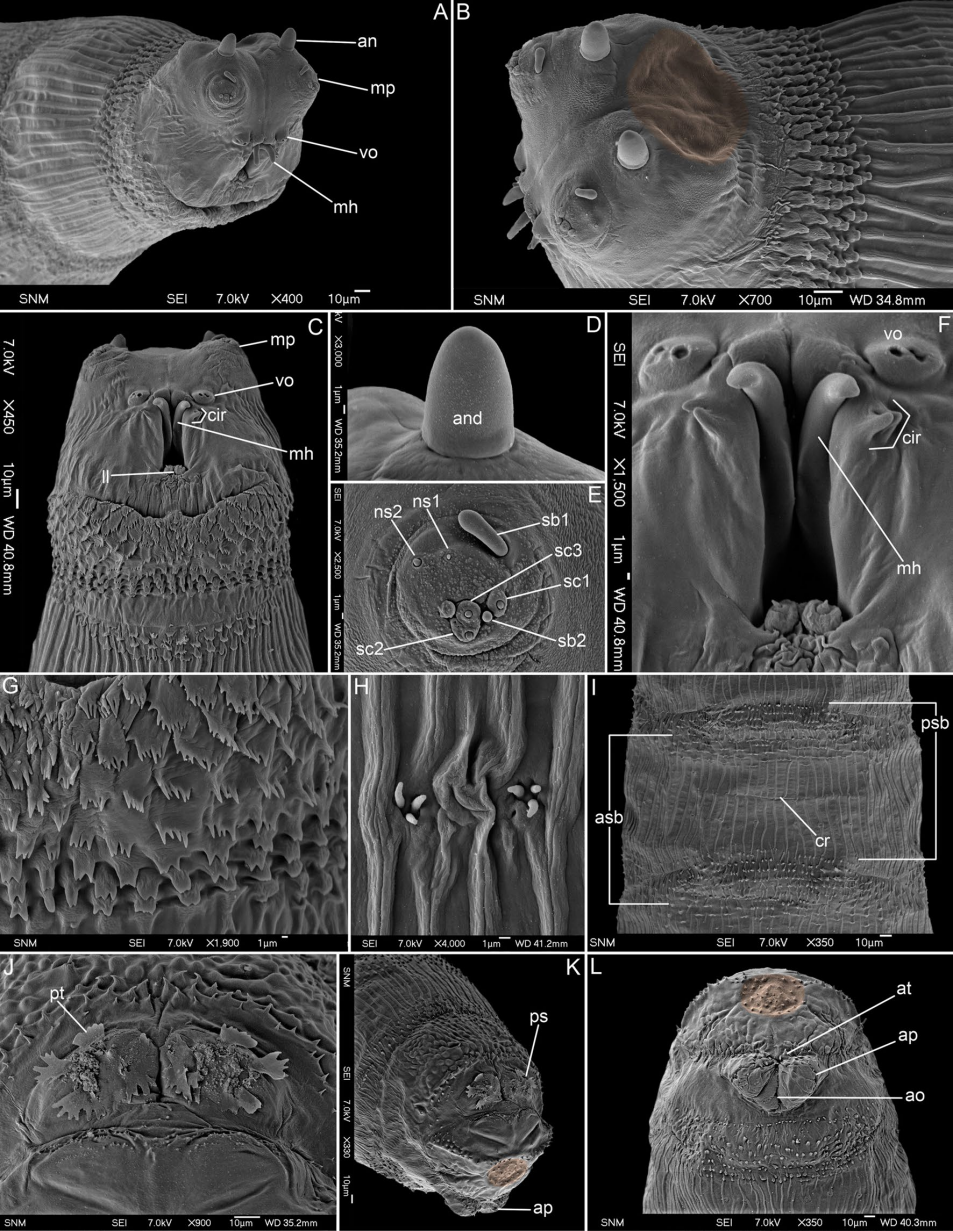
First instar larva of *Protomiltogramma cincta*. (**A**) Anterior end, antero-lateral view. (**B**) Anterior end, dorsolateral view. (**C**) Anterior end, ventral view. (**D**) Antennal complex. (**E**) Maxillary palpus. (**F**) Mouth opening. (**G**) First thoracic segment, ventral view, anterior spinose band. (**H**) First thoracic segment, Keilin’s organ. (**I**) Second abdominal segment, ventral view. (**J**) Posterior spiracles. (**K**) Posterior end, postero-lateral view. (**L**) Posterior end, ventral view. Circular depression on pseudocephalon and anal division is highlighted in brown. an, antennal complex; and, antennal dome; ao, anal opening; ap, anal papilla; asb, anterior spinose band; at, anal tuft; cir, cirri; cr, transverse crevice; ll, labial lobe; mh, mouthhook; mp, maxillary palpus; ns1, first additional sensillum coeloconicum; ns2, second additional sensillum coeloconicum; ps, posterior spiracle; psb, posterior spinose band; pt, peristigmatic tuft; sb1–3, sensilla basiconica 1–3; sc1–3, sensilla coeloconica 1–3; vo, ventral organ.

*Protomiltogramma cincta* Townsend, 1916.

(Figs. 3A–L, 5E,G, 6E, F).

Material examined: 21 larvae from a single female: /Australia, NT/Kakadu NP/Wildman River Creek/-12.82284, 132.02455/7 Nov 2017/leg. FG-NT Expedition//579 P. cincta/larva/.

*Pseudocephalo*n. Antennal complex (an) large, antennal dome (and) oval, with rounded tip, antennal basal ring entirely reduced (Fig. 3A,B,D); maxillary palpus (mp) shaped as a flat disc clearly distinguished from surrounding cuticle, first sensillum basiconicum (sb1) long with slightly swollen tip and shifted far away from central cluster of sensilla almost to medio-dorsal border of palpus (Fig. 3E); ventral organ (Fig. 3A,C,F) on small protuberance; oral ridges absent (Fig. 3A,C); pseudocephalon postero-dorsally (approximately behind base of antenna) with large, circular depression (Fig. 3B).

*Cephaloskeleton*. Labrum (lb) elongated, slender anterior part of labrum shorter than broader basal part, anterior part of labrum curved downward and with pointed tip (Figs. 5G, 6E,F); mouthhooks long and massive, middle part of mouthhooks with large process directed ventrally, tip of mouthhooks curved downward with single pointed tip (Figs. 5G, 6E,F); intermediate sclerite (is) partly hidden behind parastomal bars (pb) in lateral view (Figs. 5G, 6E,F); parastomal bar long (Figs. 5G, 6E); vertical plate (vp) with similar width like ventral and dorsal cornua (dc and vc) (Figs. 5G, 6E); dorsal bridge absent.

*Thoracic segments*. Anterior spinose band on t1 with 6–7 (dorsally) to 8–11 (ventrolaterally) rows of spines (Fig. 3A–C), laterally and ventrally the anterior spines (first 5–6 rows) flat and serrated with multicuspid tips (with 3–11 cusps) (Fig. 5G), spines situated more posteriorly (from rows 5–6 to row 11) more narrow and with single tip or with 2–3 cusps (Fig. 3G), t1 ventrally behind anterior spinose band with additional, narrow (2–3 rows) spinose band (Fig. 3C), anterior spinose bands on t2 and t3 with elongated spines, mostly with single, blunt tips; remaining area of thoracic segments with regularly set cuticular ridges (Fig. 3A,B,D); Keilin’s organ with slightly elongated sensilla (Fig. 3H).

*Abdominal segments*. Anterior spinose bands (asb) on a1–a7 with from 1–2 to 8–9 rows of spines, all bands complete (Fig. 5E), posterior spinose band (psb) on all segments complete but narrow with 1–3 rows of spines, spines small and arranged separately from each other (Fig. 3I); lateral creeping welts (lcw) developed and covered by sparse spines; all abdominal segments with regular cuticular ridges on entire surface (Fig. 3I).

*Anal division*. Anterior spinose band on anal division (ad) incomplete, without spines laterally on the lateral surfaces (Fig. 3K,L); anal division dorsally with irregular cuticular ridges and warts (Fig. 3J,K), remaining surfaces mostly with fine cuticular ridges (Fig. 3K,L); papillae around spiracular field small and visible as conical protuberances with an apical sensillum; spiracular field ringed by irregular circle of spines; below posterior spiracles transverse strip of small spines directed toward anterior end of body (Fig. 3K); posterior spiracles (ps) with four peristigmatic tufts each with numerous (5–7) branches (Fig. 3J); anal division posteriorly between spiracular field and anal complex with circular depression (Fig. 3K,L); anal papillae (ap) rounded (Fig. 3L); anal tuft (at) with several spines (Fig. 3L).

*Protomiltogramma plebeia* Malloch, 1930.

(Figs. 4A–K, 5F,H).

**Figure 4:**
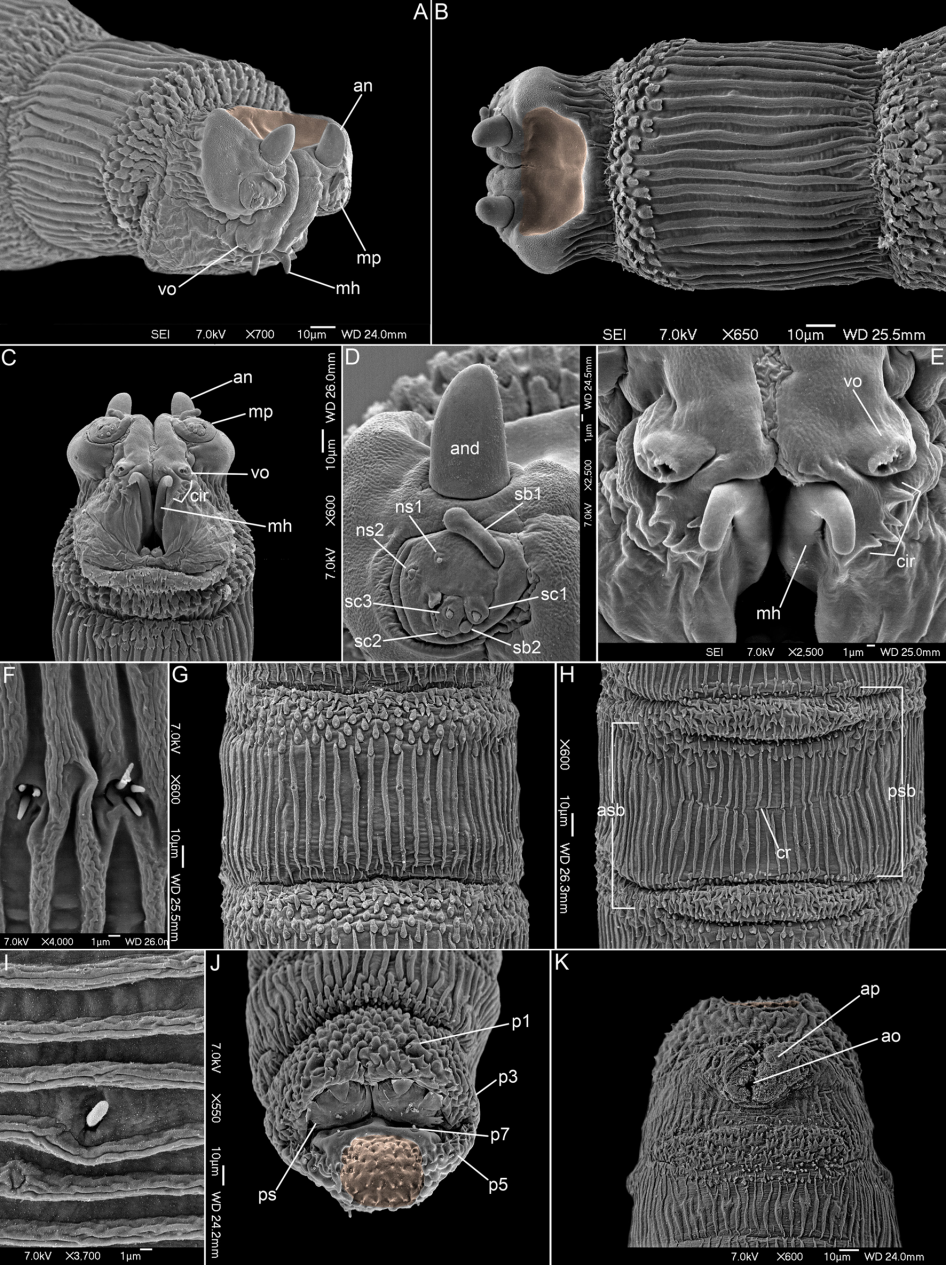
First instar larva of *Protomiltogramma plebeia*. (**A**) Anterior end, antero-lateral view. (**B**) Anterior end, dorsal view. (**C**) Anterior end, ventral view. (**D**) Antennal complex and maxillary palpus. (**E**) Mouth opening. (**F**) First thoracic segment, Keilin’s organ. (**G**) Second abdominal segment, dorsal view. (**H**) Second abdominal segment, ventral view. (**I**) Third abdominal segment, lateral papilla. (**J**) Posterior end, postero-dorsal view. (**K**) Posterior end, ventral view. Circular depression on pseudocephalon and anal division is highlighted in brown. an, antennal complex; and, antennal dome; ao, anal opening; ap, anal papilla; asb, anterior spinose band; cir, cirri; cr, transverse crevice; mh, mouthhook; mp, maxillary palpus; ns1, first additional sensillum coeloconicum; ns2, second additional sensillum coeloconicum; p1–p7, papillae around spiracular field; ps, posterior spiracle; psb, posterior spinose band; sb1–3, sensilla basiconica 1–3; sc1–3, sensilla coeloconica 1–3; vo, ventral organ.

**Figure 5:**
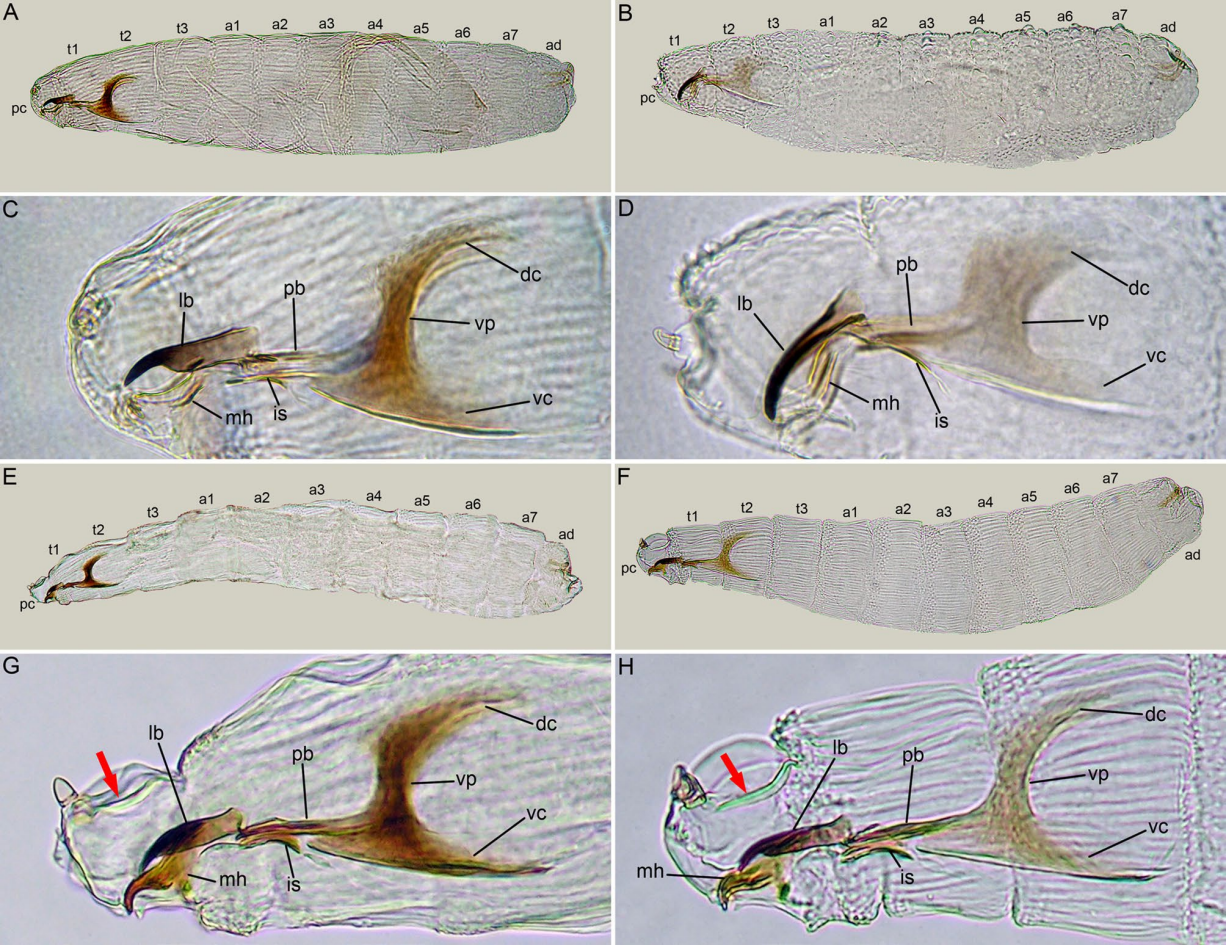
First instar larva of Australian Miltogramminae. (**A**) *Aenigmetopia amissa*, habitus, lateral view. (**B**) *Amobia auriceps*, habitus, lateral view. (**C**) *Aenigmetopia amissa*, cephaloskeleton, lateral view. (**D**) *Amobia auriceps*., cephaloskeleton, lateral view. (**E**) *Protomiltogramma cincta*, habitus, lateral view. (**F**) *Protomiltogramma plebeia*, habitus, lateral view. (**G**) *Protomiltogramma cincta*, cephaloskeleton, lateral view. (**H**) *Protomiltogramma cincta*, cephaloskeleton, lateral view. Scale bar 0.1 mm. a1–7, abdominal segments; ad, anal division; dc, dorsal cornua; is, intermediate sclerite; lb, labrum; mh, mouthhook; pb, parastomal bar; pc, pseudocephalon; t1–3, thoracic segments; vc, ventral cornua; vp, vertical plate.

Material examined: 16 larvae from a single female: /Australia, NT, ‘590’/West MacDonnell NP/Serpentine Gorge/-23.75686 132.9814/13 Nov 2017/leg. FG-NT Expedition/.

*Pseudocephalon*. Antennal complex (an) very large, antennal dome (and) oval, with slightly conical tip, antennal basal ring entirely reduced (Fig. 4A–D); maxillary palpus (mp) shaped as a flat disc clearly distinguished from surrounding cuticle, first sensillum basiconicum (sb1) long with slightly swollen tip and shifted far away from central cluster of sensilla almost to medio-dorsal border of palpus (Fig. 4D); ventral organ (vo) (Fig. 4A,C,E) on small protuberance; oral ridges absent (Fig. 4A,C,E); pseudocephalon postero-dorsally (approximately behind base of antenna) with large, circular depression (Fig. 4A,B).

*Cephaloskeleton*. Labrum (lb) elongated, slender anterior part of labrum shorter than broader basal part, anterior part of labrum curved downward and with pointed tip (Fig. 5H); mouthhooks long and massive, middle part of mouthhooks with large process directed ventrally, tip of mouthhooks curved downward with single pointed tip (Fig. 5H); intermediate sclerite (is) partly hidden behind parastomal bars (pb) in lateral view (Fig. 5H); parastomal bar long (Fig. 5H); vertical plate (vp) slightly wider than ventral and dorsal cornua (dc and vc) (Fig. 5H); dorsal bridge absent.

*Thoracic segments*. Anterior spinose band on t1 with 5–6 (dorsally) to 6–11 (ventro-laterally) rows of spines (Fig. 4A–C), laterally and ventrally the anterior spines (to 5–6 row) flat and serrated with multicuspid tips (with 3–11 cusps) (Fig. 5G), spines situated more posteriorly (from rows 5–6 to row 11) more narrow and with single tip or with 2–3 cusps (Fig. 4G), t1 ventrally behind anterior spinose band with additional, narrow (2–3 rows) spinose band (Fig. 4C), anterior spinose bands on t2 and t3 with elongated spines, mostly with single, blunt tips (Fig. 4B); remaining area of thoracic segments with regularly set cuticular ridges (Figs. 4A–C, 5F,H); Keilin’s organ with slightly elongated sensilla (Fig. 4F).

*Abdominal segments*. Anterior spinose bands (asb) on a1–a7 with from 1–2 to 8–9 rows of spines, all bands complete (Figs. 4G,H, 5F), posterior spinose band (psb) present complete only on a5–a7, narrow with 1–3 rows of spines, spines small and arranged separately from each other (Fig. 4G,H); lateral creeping welts (lcw) developed and covered by sparse spines; all abdominal segments with regular cuticular ridges on entire surface (Figs. 4G–I, 5F).

*Anal division*. Anterior spinose band on anal division (ad) incomplete, without spines laterally (Fig. 4J,K); anal division dorsally with irregular cuticular warts (Fig. 4J), ventral surface until anal opening with fine cuticular ridges (Fig. 4K), remaining surface of anal division with irregular pattern of ridges, warts and spines (Fig. 4J); papillae around spiracular field small and visible as conical protuberances with an apical sensillum (Fig. 4J); spiracular field ringed by irregular circle of spines (Fig. 4J); posterior spiracles (ps) with four peristigmatic tufts each with numerous (5–7) branches (Fig. 4J); anal division posteriorly between spiracular field and anal complex with circular depression (Fig. 4J,K); anal papillae rounded (Fig. 4K); anal tuft (at) with several spines.

## Discussion

The first molecular-based reconstruction of the phylogeny of the Miltogramminae enabled a formal testing of the hypotheses for the evolution of the larval morphology and feeding strategies of this taxon^1^. The ancestor of the Miltogramminae was likely a saprophage specialised for utilising vertebrate carrion buried in the ground, as highlighted by the combination of its large pseudocephalic sensory organs and well-developed cuticular sculpture, along with retention of a plesiomorphic dorsal bridge in the cephaloskeleton^1^. Larvae of Australian species, including those of the endemic *Aenigmetopia*, share synapomorphies such as the loss of a dorsal bridge and hair-like spines around the spiracular field with other kleptoparasitic Miltogramminae. This evidence supports their position within the ‘higher’ miltogrammines sensu Piwczyński et al.^1^.

While the morphology of first instars of *Am. auriceps*, *P. cincta* and *P. plebeia* is generally similar to that of their Palaearctic relatives, the first instar of *Ae. amissa* appears to be the most intriguing. Hypotheses about the phylogenetic affinities of the genus *Aenigmetopia* have been proposed several times. Rohdendorf^15^ placed this genus in the tribe Senotainiina, together with *Senotainia* and *Eumacronychia* Townsend. Subsequently, Verves^5^ transferred *Aenigmetopia* to the tribe Miltogrammatini as sister to the subtribe Pterellina, comprising the genera *Chaetapodacra* Rohdendorf, *Eremasiomyia* Rohdendorf, *Pterella* Robineau-Desvoidy and *Protomiltogramma*. Most recently, Johnston et al.^9,12^ presented molecular data in support of a sister-group relationship between *Aenigmetopia* and a subset of *Metopia*, rendering the latter genus paraphyletic. While this conflicts with the traditional morphological circumscription of *Metopia*^6^, it may be considered to constitute the best-supported hypothesis for the phylogenetic position of *Aenigmetopia* to date, even if the support is low (BS = 60, PP = 0.84)^12^. An interesting similarity is observed between *Ae. amissa* and *Senotainia conica* (Fallén)^2^, which share the following character states: (1) antennal complex with high basal ring, (2) club-like first sensillum basiconicum situated close to the central cluster of sensilla, (3) ventral organ sensilla placed on a small fleshy lobe, (4) oral ridges present, (5) mouthhook with serrated tip, and (6) cuticular sculpture in the form of regular ridges. However, most of these character states cannot be considered to provide a strong phylogenetic signal as they are also present in taxa situated in different clades of the miltogrammine phylogenetic tree, and they are symplesiomorphic at this level^1,2,9,12^. Additionally, the shape of the oral ridges and the general morphology of the anal division of *Ae. amissa* and *S. conica* are different, but the autapomorphic condition in *Ae. amissa* makes phylogenetic deductions premature. Searching for larval synapomorphies between *Aenigmetopia* and *Metopia* is made complicated by the diverse larval morphology of *Metopia*^2,16^. Despite this, *Ae. amissa* and the five species of *Metopia* for which larvae have been described to date (*M. argentata* Macquart, *M. argyrocephala* (Meigen), *M. campestris* (Fallén), *M. grandii* Venturi, *M. italiana* Pape) share a uniquely shaped apical part of the mouthhook, which is serrated with multiple teeth orientated perpendicular to the main axis of the mouthhook^2,16^. Additionally, the mouthhook in *Ae. amissa* has teeth with rounded tips, which is a character state expressed in all species of *Metopia* except *M. campestris*. Overall, the morphological similarities between *Ae. amissa* and *Metopia* spp. support a monophylum consisting of *Aenigmetopia* and *Metopia* as suggested by Johnston et al.^12^. Unfortunately, the first instar larva of *M. nudibasis*, the sister species to *Aenigmetopia* in the phylogenetic tree provided by Johnston et al.^12^, remains unknown. Therefore, the possible paraphyly of *Metopia* cannot be tested by larval morphology, and formal synonymization of *Aenigmetopia* under *Metopia* is considered premature and should await further analyses based on additional taxa and more data.

The first instar of *Am. auriceps* is almost identical to those of the Palaearctic species in most characters^2^, including: (1) long and slender first sensillum basiconicum situated close to the central cluster of sensilla, (2) ventral organ sensilla placed on a large fleshy lobe, (3) oral ridges short, (4) labrum long, curved downward, gradually tapering to pointed anterior tip, (5) broad basal part of mouthhook, (6) cuticular sculpture on abdominal segments in the form of warts, (7) abdominal segments ventrally with locomotory protuberances/prolegs, (8) abdominal segments dorsally with transverse row of large triangular cones, (9) peristigmatic tufts small, and (10) spiracular field without encircling spines. The first instar of *Am. auriceps* differs from the majority of Palaearctic *Amobia* in two key characters, which henceforth should be considered to be intragenerically variable: (1) the antennal basal ring of *Am. auriceps* more pronounced than the low and flat structure described in the larvae of Palaearctic species; and (2) the entire lateral surface of thoracic segments in *Am. auriceps* covered by cuticular warts (Fig. 2B), whereas at least in the Palaearctic *Amobia* the lateral surface of the first thoracic segment is covered by dense cuticular ridges^2^.

The first instar larva of species representing the genera *Eremasiomyia*, *Protomiltogramma* and *Pterella* share the following set of character states^2,3^: (1) basal ring in antennal complex absent, (2) first sensillum basiconicum shifted to the inner periphery of maxillary palpus, and (3) posterior surface of anal division between spiracular field and anal complex with circular depression. This set of features is not observed in larvae of other miltogrammines, and *Eremasiomyia*, *Protomiltogramma* and *Pterella* probably form a monophylum. The morphology of the first instar larvae of Australian *Protomiltogramma* is almost identical to that of the Palaearctic species *P. fasciata* (Meigen) in all aspects, such as the form and distribution of the sensory organs on the pseudocephalon, the cephaloskeleton, the cuticular sculpture and the shape of the anal division. The presence of a large circular depression on the pseudocephalon and on the anal division represent two intriguing character states of *Protomiltogramma* larvae. Szpila^2^ did not mention these depressions in his description of the larva of *P. fasciata*, but they are plainly visible in his Fig. 56, and they have also been observed in larval material of *Protomiltogramma* from the United Arab Emirates (K. Szpila, unpubl.). These depressions have been described only in the genera *Protomiltogramma* (three species, depressions on both pseudocephalon and anal division), *Pterella* Robineau-Desvoidy (two species, depression only on anal division) and *Phrosinella* Robineau-Desvoidy (three species, depression only on anal division)^1,2,17^. They are of a very similar morphology and therefore unlikely to be artefacts. However, the function of these circular depressions remains unknown and needs further study using other approaches, e.g. anatomical methods.

The present study fills an important gap in our knowledge both of the preimaginal instars of miltogrammine flies and the morphological diversity of endemic Australian taxa. Congruence was established between larval morphology and the current phylogenetic hypotheses presented for the Australian Miltogramminae^9,12^ and the global Miltogramminae^1,18^. This alignment between larval morphology and molecular phylogeny in the Australian Miltogramminae provides evidence for both the stability of these traits in the Sarcophagidae and their potential value for the inference of evolutionary relationships in this family.

## Methods

Adult female miltogrammine flies were collected from various locations in the Northern Territory, Australia (details available in the “Results”). Once preliminarily identified, freshly caught females were killed with ethyl acetate vapour and transferred to a stereomicroscope. Live first instar larvae were then obtained by gently squeezing the female’s abdomen. Following larval extraction, females were either pinned (*Protomiltogramma*) or preserved in 95% ethanol (*Aenigmetopia*, *Amobia*). Females were identified using the keys of Malloch^19^, Johnston^8^ and Johnston et al.^9,11^ in combination with comparative studies of voucher specimens from the Australian National Insect Collection, Canberra, Australia (ANIC); the Australian Museum, Sydney, Australia; the Department of Ecology and Biogeography, Nicolaus Copernicus University, Toruń, Poland (DEB); and the Natural History Museum of Denmark, University of Copenhagen, Denmark.

Larvae were killed by soaking in hot water (about 95 °C) to avoid deformation and stored in 70% ethanol. Preserved larvae were slide-mounted in Hoyer’s medium for light microscopy. Preparation for scanning electron microscopy (SEM) involved dehydration through 80, 90 and 99.5% ethanol and critical-point drying in CO_2_, after which the larvae were coated with ~ 100 nm of platinum/palladium. SEM images were taken with the use of a JEOL JSM 6335F field emission microscope. Light microscope illustrations were produced from photographs made with the use of a digital Nikon 8400 camera mounted on a Nikon Eclipse E200 microscope.

Material intended for visualization with confocal laser scanning microscopy (CLSM) was freshly prepared from whole available larvae. Specimens were cleared initially by immersion in 10% potassium hydroxide for over 48 h, until tissue digestion was satisfactorily obtained. The material was then dehydrated in 99.5% EtOH for 20 min and the specimens were embedded in Euparal on a microscope slide and covered with a coverslip. Due to the small size of the examined material, flat microscope slides were used to avoid damaging the structures of interest. Prepared slides were scanned using a Leica TCS SP8 Confocal Laser Scanning Microscope. In order to obtain optimal fluorescence, only the excitation wavelengths of 561 nm and 633 nm were used. A strong fluorescence signal was also obtained with an excitation wavelength of 488 nm, but this was omitted as it simultaneously revealed some contamination and undigested tissue, which significantly disturbed the resulting image. First instars of *Ae. amissa* and *Am. auriceps* were similar in size and examined under a 63× objective lens with a numerical aperture (NA) of 1.4, and the first instar of *P. cincta* was examined under 40× with a NA of 1.3. Effective 3D visualization requires collecting more data than necessary to obtain acceptable 2D images^20^, and differences in the thickness of the individual larvae required optimization of the amount of data collected from each frame. Individual z-step size settings were defined for each sample manually. The larger size in the z-plane imposed the collection of fluorescence signals from different focal planes, which allowed for full visualization of the examined structures. After the acquisition of the preliminary image series, the 3D model was built in the LAS X 3D Viewer program. When the received model was of low quality acquisition of image series was repeated with manually increased number of z-steps. The respective number of z-frames was individually adjusted for each of the three specimens; for *Ae. amissa* there were 123 z-frames, for *Am. auriceps* 146 z-frames and for *P. cincta* 224 z-frames.

**Figure 6:**
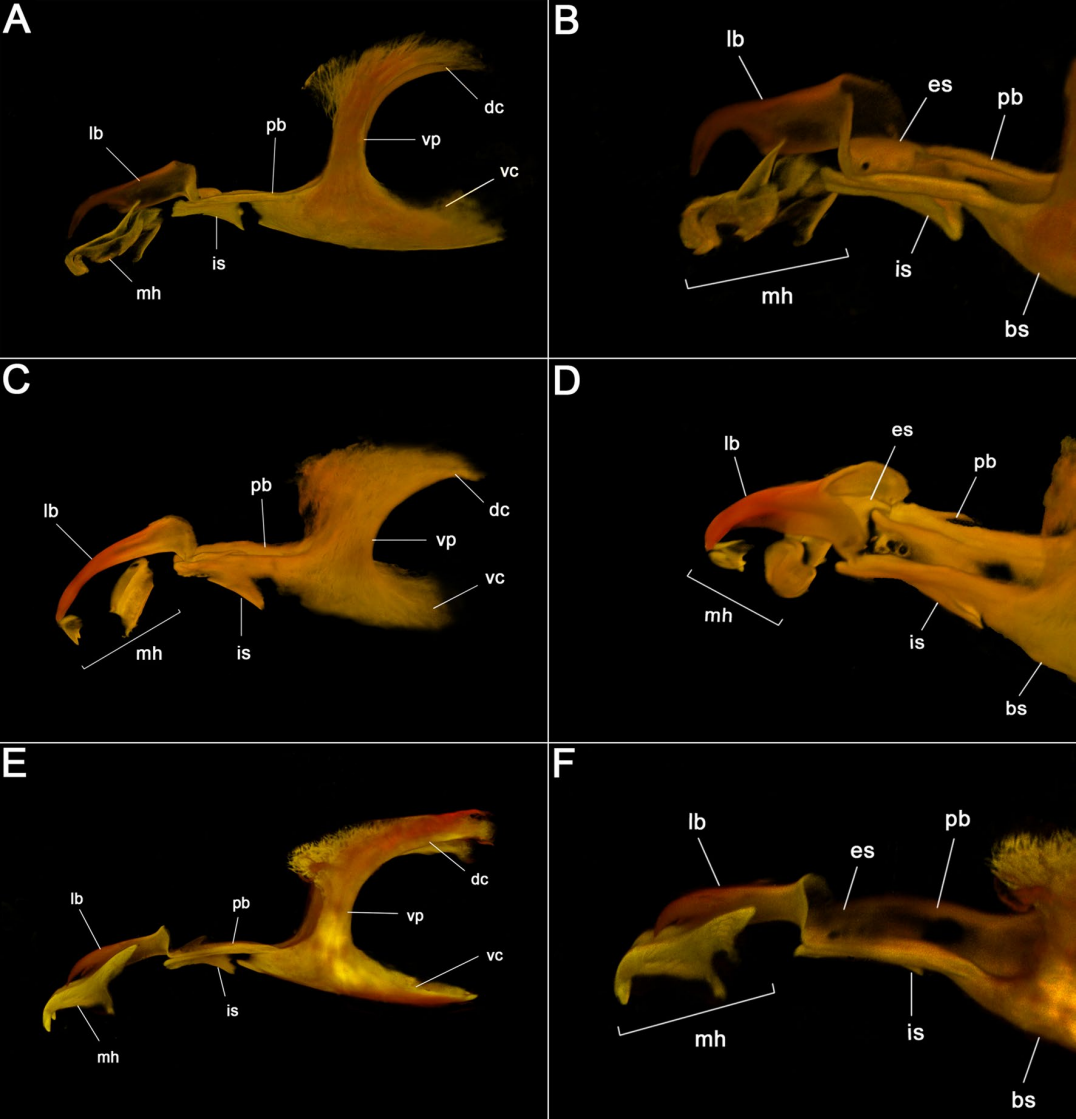
CLSM images of first instar larva of Australian Miltogramminae (**A**) *Aenigmetopia amissa*, cephaloskeleton, lateral view. (**B**) *Aenigmetopia amissa*, anterior part of cephaloskeleton, postero-lateral view. (**C**) *Amobia auriceps*, cephaloskeleton, lateral view. (**D**) *Amobia auriceps*, anterior part of cephaloskeleton, postero-lateral view. (**E**) *Protomiltogramma cincta*, cephaloskeleton, lateral view. (**F**) *Protomiltogramma cincta*, habitus, anterior part of cephaloskeleton, postero-lateral view. dc, dorsal cornua; es, epistomal sclerite; is, intermediate sclerite; lb, labrum; mh, mouthhook; pb, parastomal bar; vc, ventral cornua; vp, vertical plate.

The females of *P. cincta* and *P. plebeia*, all larvae, and associated microscope slides have been deposited in DEB. Females of *Ae. amissa* and *Am. auriceps* have been deposited in ANIC. Larval terminology follows Courtney et al.^21^, with a few modifications proposed by Szpila and Pape^22^ and Szpila^2^. Nomenclature follows Pape^6^ and Johnston et al.^9,11^, with the generic names of *Aenigmetopia* and *Amobia* abbreviated as *Am*. and *Ae*., respectively, to avoid confusion. Label data of specimens are given verbatim using a slash (/) for the start and end of a line, and a double slash (//) for the end of a label and the beginning of the next (from top to bottom on the same pin) (Fig. 6).
